# Genome-Wide Identification and Characterization of *TCP* Genes in Eight *Prunus* Species and Their Expression Patterns Under Cold Stress in *P. tenella* var. *tenella*

**DOI:** 10.3390/genes15111443

**Published:** 2024-11-08

**Authors:** Qiang Zhang, Cheng Qian, Lulu Li, Wei Li, Yanhua Li, Han Zhao

**Affiliations:** 1 Institute of Marine Science and technology, Shandong University, Qingdao 266215, China; qusdream@163.com; 2 College of Landscape Architecture and Forestry, Qingdao Agricultural University, Qingdao 266109, China; 20222110027@stu.qau.edu.cn (C.Q.); yuanlinlilulul@126.com (L.L.); weili@qau.edu.cn (W.L.); 3 Research Institute of Non-Timber Forestry, Chinese Academy of Forestry, Zhengzhou 450014, China

**Keywords:** cold stress, expression pattern, *Prunus* genus, *Prunus tenella* var. *tenella*, Teosinte branched1/Cycloidea/Proliferating cell nuclear antigen factors

## Abstract

Background/Objectives: Teosinte branched1/Cycloidea/Proliferating cell nuclear antigen factors (TCPs) are plant-specific transcription factors involved in leaf development, flowering, branching, hormone signaling, and stress responses. Prunus a key temperate fruit tree with ornamental spring blooms, still lacks comprehensive TCP gene studies across many species. Methods: We identified 154 TCP genes in eight Prunus species: 19 in *Prunus tenella* var. *tenella*, 19 in *P*. *amygdalus*, 17 in *P*. *armeniaca* ‘Rojo Pasion’, 19 in *P*. *mira*, 20 in *P*. *jamasakura* var. *jamasakura*, 19 in *P*. *fruticosa*, 19 in *P*. *mume* var. *tortuosa*, and 22 in *P*. × *yedoensis* ‘Somei-yoshino’. These genes were classified into PCF, CIN, and CYC/TB1 groups. We examined segmental duplication, conserved motifs, and cis-acting elements. Expression patterns of 12 TCPs in *P*. *tenella* var. *tenella* were tested under low-temperature stress (25 °C, 5 °C, −5 °C, and −10 °C), and *PtTCP9*’s subcellular localization was determined. Results: TCP genes within the same groups showed similar motifs and cis-acting elements. Cold stress analysis identified multiple low-temperature-responsive elements in gene promoters. Four genes (*PtTCP2*, *PtTCP6*, *PtTCP14*, and *PtTCP16*) increased expression under cold stress, while six genes (*PtTCP1*, *PtTCP5*, *PtTCP8*, *PtTCP9*, *PtTCP17*, and *PtTCP19*) decreased. *PtTCP9* was localized to the nucleus. Conclusions: This was the first genome-wide study of the TCP gene family in these eight *Prunus* species, providing valuable insights into the characteristics and functions of TCP genes within this important genus.

## 1. Introduction

Transcription factors play a crucial role in plant growth, development, and response to environmental stress. TCP is a plant-specific transcription factor family named after its first characterized members: Teosinte branched1 from maize (*Zea mays*), Cycloidea from *Antirrhinum*, and PCF from rice (*Oryza sativa*) [1,2,3]. Sequence alignment analysis has revealed that members of the *TCP* gene family contain a unique domain known as the TCP domain, which features a nonclassical basic helix–loop–helix structure. This domain is mainly related to DNA binding, protein interaction, and protein nuclear localization [1,2,3,4]. The nonclassical bHLH (basic Helix–Loop–Hleix) motifs are located at the N-end of the TCP domain [1,2,3,5]. The TCPs from eight *Prunus* species were classified into Class I and Class II subfamilies. Class I is also known as the PCF subfamily, and Class II is further divided into CIN and CYC/TB1 groups. The R domain is found only in members of the CYC and TB1 subfamilies and plays a role in protein interactions [4].

The TCP gene family plays key roles in plant growth, development, and response to stresses, influencing processes such as seed germination, bud growth, flower organ development, leaf morphogenesis, apical dominance, axillary meristem development [6,7,8,9,10,11], and hormonal signal transduction [12,13]. TCP members can be regulated by endogenous signals such as plant hormones [14]. The functions of TCP proteins in the cold resistance of plants have been identified [15,16]. In *O. sativa*, the overexpression of *OsTCP14* and *OsTCP21* increases the sensitivity of plants to low temperatures, and silencing these two TCP genes through RNAi technology enhances low-temperature tolerance in *O. sativa* [17]. In addition, TCP members can respond to exogenous factors such as abiotic stress [18].

The *Prunus* genus comprises more than 200 species. Many *Prunus* plants are important fruit and nut crops as well as ornamental plants. Although various classification systems exist for *Prunus* species, the most widely accepted is Rehder’s classification, which divides this genus into five subgenera: *Prunus*, *Amygdalus*, *Padus*, *Cerasus*, and *Laurocerasus* [19]. *P. mume* and *P. armeniaca*, which belong to the *Prunus* subgenus, are fruit crops and ornamental trees in East Asia. *P. jamasakura* var. *jamasakura*, *P.* × *yedoensis*, and *P. fruticosa* belong to the *Cerasus* subgenus and are important ornamental woody plant that bloom in spring. *P. fruticosa* is a wild species in the temperate regions of Europe and Asia [20]. Studies have shown that *P. fruticosa* is one of the parents of cherry (*P. cerasus*) [21]. *P. amygdalus*, *P. tenella* var. *tenella*, and *P. mira* belong to the *Amygdalus* subgenus. *P. amygdalus* is an important nut crop and ornamental tree. *P. tenella* var. *tenella* is a related species of almond and is also known as the wild almond [22]. It is an excellent raw material for breeding new resistant varieties of almond and drought- and cold-resistant dwarf rootstock types of stone fruit trees [23,24,25]. TCP family members have been identified in many plants, excluding the aforementioned species.

This study aims to identify TCP family genes of eight species from three subgenera of *Prunus*. The phylogenetic analysis, conserved motifs, sequence alignment, chromosome localization, collinearity analysis, and cis-acting element analysis of TCP genes were conducted. Additionally, the study sought to investigate the expression pattern of *PtTCPs* under cold stress using quantitative real-time polymerase chain reaction (qRT-PCR) and to verify the subcellular localization of the *PtTCP9* transcription factor. The overall goal was to preliminarily reveal the evolutionary correlation of the TCP family in *Prunus* and the gene expression changes in response to cold stress in *P. tenella* var. *tenella*.

## 2. Materials and Methods

### 2.1. Taxon Sampling

The genomes of *P. amygdalus* (GCA_902201215.1), *P. armeniaca* ‘Rojo Pasion’ (GCA_903112645.1), *P. fruticosa* (GCA_018703695.1), *P. jamasakura* var. *jamasakura* (GCA_020521455.1), *P. mira* (GCA_020226265.1), *P. mume* var. *tortuosa* (GCA_029339155.1), and *P. × yedoensis* ‘Somei-yoshino’ (GCA_005406145.1) were downloaded from the NCBI website. The whole genome sequence data of the *P. tenella var. tenella* genome have been deposited in the Genome Warehouse at the National Genomics Data Center (accession number: GWHCBGA00000000). *P. tenella* var. *tenella* seeds were collected from Toli County, Xinjiang, China (46°08′51.33″ N; 83°33′56.33″ E; Elevation 823 m), and the characteristics of *P. tenella* var. *tenella* are shown in Figure S1. Then the seeds were treated with a solution containing gibberellin (200 mM) for 24 h to release dormancy, after which they were planted in plastic pots (15 × 12 × 14 cm) with consistent conditions (22 °C, 16 h light/8 h dark). For the convenience of taxonomic sampling and discussion, we have used and applied the names of species and infraspecific taxa according to POWO (https://powo.science.kew.org/, accessed on 28 October 2024), which includes the latest classifications and information on global plant species.

### 2.2. Identification of TCP Genes

The TCP Hidden Markov model (PF03634) downloaded from the Pfam website (http://pfam.xfam.org/, accessed on 2 July 2024) was run on the whole-genome protein sequence of eight *Prunus* species (*P. tenella* var. *tenella*, *P. amygdalus*, *P. armeniaca* ‘Rojo Pasion’, *P. fruticosa*, *P. jamasakura* var. *jamasakura*, *P. mira*, *P. mume* var. *tortuosa*, *P.* × *yedoensis* ‘Somei-yoshino’). The obtained sequence was used to build the TCP Hidden Markov model, and a search was performed again with this model to obtain members of the TCP family. The Conserved Domains Tool and InterProScan v102.0 (https://www.ebi.ac.uk/interpro/, accessed on 2 July 2024) online tools were used to confirm the conserved domains in protein sequences of TCPs, and the proteins that did not contain TCP domains were removed.

### 2.3. Chromosomal Location and Structure Analysis of TCPs

The distribution of TCPs on chromosomes was plotted using the gff3 file from genomes and TBtools-II v2.085 software [26]. An online analysis of conserved motifs of different TCP proteins was conducted using MEME v5.5.6 (https://meme-suite.org/meme/, accessed on 29 August 2024) [27], with a motif search number of 10. The obtained motifs were visualized and analyzed using TBtools software.

### 2.4. Prediction of cis-Regulatory Elements and Transcription Start Sites in the Promoter of PtTCPs

TBtools software was used to extract the 2000 bp upstream sequence of ATG as the promoter subsequence [26]. At the same time, the PlantCARE v1.0 Database (https://bioinformatics.psb.ugent.be/webtools/plantcare/html/, accessed on 20 August 2024) was used to predict the type and number of cis-regulatory elements in each member’s promoter. The data were screened and plotted in Excel 2016.

### 2.5. Construction of Phylogenetic Tree 

We downloaded the genomes of *Arabidopsis thaliana* and *O*. *sativa* from NCBI, extracted TCP protein sequences as outgroups, constructed an amino acid matrix containing TCP protein sequences from eight *Prunus* species (Supplementary Data S1), and conducted phylogenetic analysis using MEGA 6.0. The tree was optimized using the neighbor-joining method, 1000 bootstrap tests, and the online tool iTOL v6.0 (https://itol.embl.de/, accessed on 22 July 2024). 

### 2.6. Cold-Stress Treatment and qPCR Analyses

To further understand the expression patterns of the *TCP* genes in *P. tenella* var. *tenella* in response to cold stress, based on previous research results, nine genes were selected: *PtTCP1*, *PtTCP2*, *PtTCP3*, *PtTCP4*, *PtTCP5*, *PtTCP6*, *PtTCP8*, *PtTCP*9, and *PtTCP12*. Two-month-old *P. tenella* var. *tenella* seedlings were transferred to an incubator, and the temperature was changed at 3 °C/h until the final target temperatures (25, 5, 0, −5, and −10 °C) were reached. All samples were immediately frozen in liquid nitrogen and stored at −80 °C for further experiments.

The total RNA was extracted from all samples using the Easy Fast reagent (Tiangen, Beijing, China). Subsequently, SuperMix (Vazyme, Beijing, China) was used to reverse transcribe RNA into cDNA. The primers were identified based on the protein sequence of the desired gene (https://www.ncbi.nlm.nih.gov/tools/primer-blast/index.cgi?LINK_LOC=BlastHome, accessed on 30 August 2024) (Table S1). Then, qPCR experiments were conducted on the selected genes. Three biological replicates were employed, with the *PtPP2A* gene as the reference gene. The 2^−ΔΔCt^ method was used to calculate the relative expression level of the *PtTCP* genes.

### 2.7. Subcellular Localization of PtTCP9 Protein

The full-length coding sequences (CDS) of the *PtTCP9* gene were cloned using the complementary DNA (cDNA) of *P*. *tenella* var. *tenella* leaf as the template, and the *PtTCP9* gene was connected to the vector 1300-GFP by the double enzyme digestion method. The primers used in the study are shown in Table S1. The overexpressed vector was transformed into DH5α *Escherichia coli* in receptor state, and the positive clones were identified by colony PCR and confirmed by sequencing. The correctly sequenced plasmid was then transformed into the Agrobacterium GV3101 in receptor state. Positive colonies were selected and expanded in Luria–Bertani (LB) liquid medium until the OD_600_ reached approximately 0.6. The infection solution (MS + 10 mM MES + 0.15 mM AS + 10 mM MgCl2) was resuspended to an OD_600_ of about 0.8 and incubated at room temperature for 2 h. The lower epidermis of *Nicotiana. benthamiana* leaves was injected with a syringe, incubated at 25 °C for 24 h, cultured in the presence of light for 24 h, and observed and photographed under a laser scanning confocal microscope (Agilent, Santa Clara, CA, USA).

## 3. Results

### 3.1. Identification and Chromosomal Location of TCP Genes

**Figure 1:**
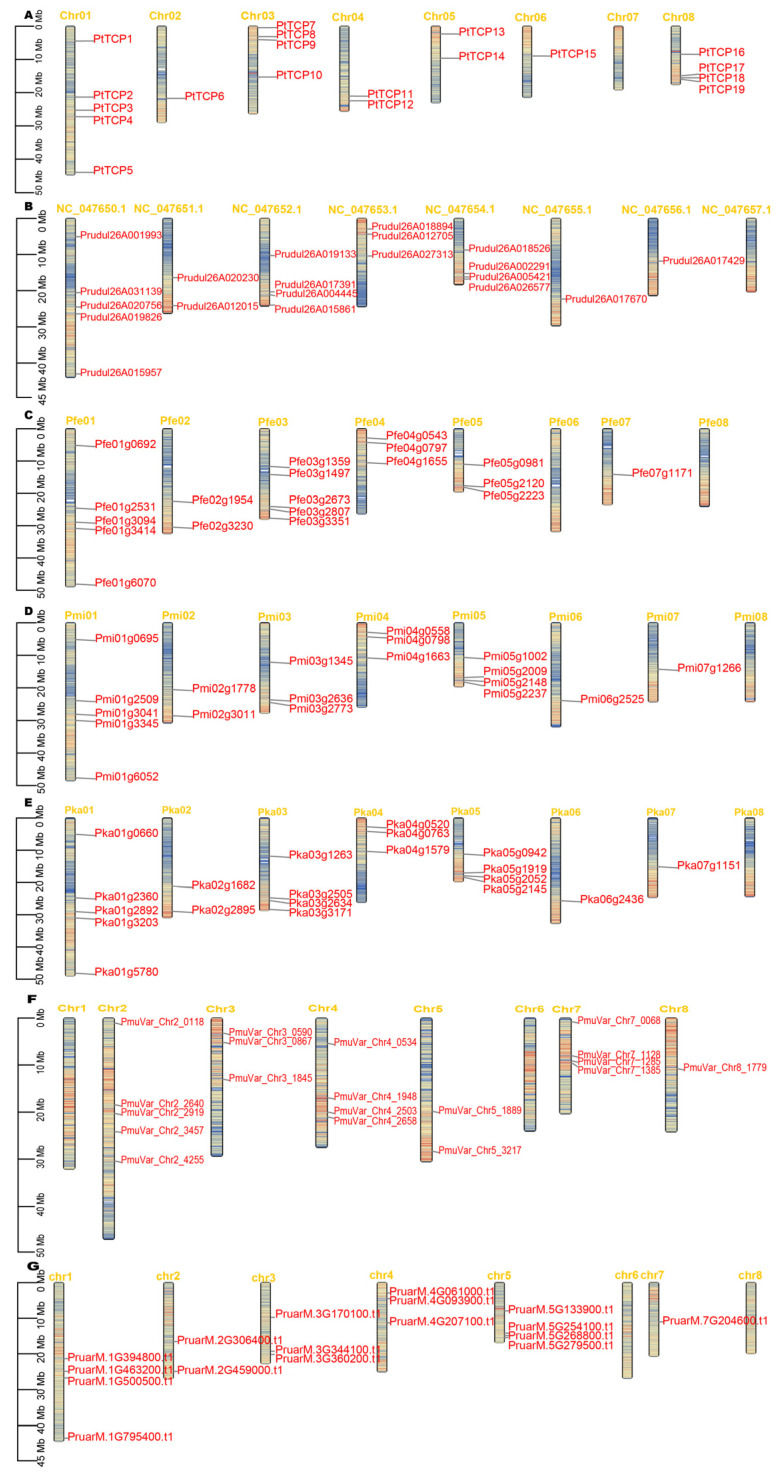
Chromosomal location of the *TCPs* in eight *Prunus* species. (**A**) *P. tenella* var. *tenella*; (**B**) *P. amygdalus*; (**C**) *P. fruticosa*; (**D**) *P. mira*; (**E**) *P. jamasakura* var. *jamasakura*; (**F**) *P. mume* var. *tortuosa*; (**G**) *P. armeniaca* ‘Rojo Pasion’. The scale (Mb) represents the length of the chromosome. Chr represents chromosomes, and the colors on the chromosomes represent gene density, with red representing high gene density and blue representing low gene density.

A total of 19, 19, 17, 19, 20, 19, 19, and 22 *TCP* genes were identified in the *P. tenella* var. *tenella*, *P. amygdalus*, *P. armeniaca* ‘Rojo Pasion’, *P. fruticosa*, *P. jamasakura* var. *jamasakura*, *P. mira*, *P. mume* var. *tortuosa*, and *P.* × *yedoensis* ‘Somei-yoshino’, respectively, using HMM maps (PF03634) (Table 1). These candidate proteins were then confirmed after further validation using the conserved domain database (CDD) and Pfam database. The TCP genes in *P. tenella* var. *tenella* were annotated as *PtTCP1* to *PtTCP19* based on their genome distribution and relative linear orders among the respective chromosomes (Figure 1). The proportion of *TCPs* ranged from 17 to 22, the highest in *P.* × *yedoensis* ‘Somei-yoshino’, followed by *P. jamasakura* var. *jamasakura* and *P. fruticosa*, while *P. armeniaca* ‘Rojo Pasion’ had the least (Table 1).

Due to variable shear, 154 genes encode 157 TCP proteins (Table 1). The length of TCP proteins varied from 145 (Pfe03g1497) to 966 (PmuVar_Chr4_1948) amino acid residues (Table S2). The molecular weight (MW) ranged from 15.0 to 104.9 kDa. The protein isoelectric point (pI) ranged from 4.63 (PtTCP12) to 11.22 (PtTCP15), with a mean of 7.23. The calculation range of the hydrophilicity index (GRAVY) values for all TCPs was between −1.015 and 0.128, with only positive values for PmuVar_Chr4_1948. This indicates that most TCPs were essentially hydrophilic. The range of the instability index was between 34.0 and 76.7. The value of the aliphatic index ranged from 51.4 to 90.9. Among those TCP proteins, 89.2% proteins were located in the nucleus, 15 TCP were on the chloroplast, and only PmuVar_Chr4_1948 and PmuVar_Chr5_3217 were found in the Plasma membrane and Cytoplasm, respectively.

Because the *P.* × *yedoensis* ‘Somei-yoshino’ genome is not assembled to the chromosomal level, chromosomal localization of their TCP genes is not possible. In seven *Prunus* species, TCP genes were unevenly distributed on 6–7 chromosomes. Except for *P. mume* var. *tortuosa*, which has no TCP gene on Chr01, the other six species have the maximum number of *TCP*s on Chr01. Except for *P. tenella* var. *tenella* and *P. mume* var. *tortuosa*, the other species have no TCP gene on Chr08.

### 3.2. Phylogenetic Analysis of TCP Proteins

**Figure 2:**
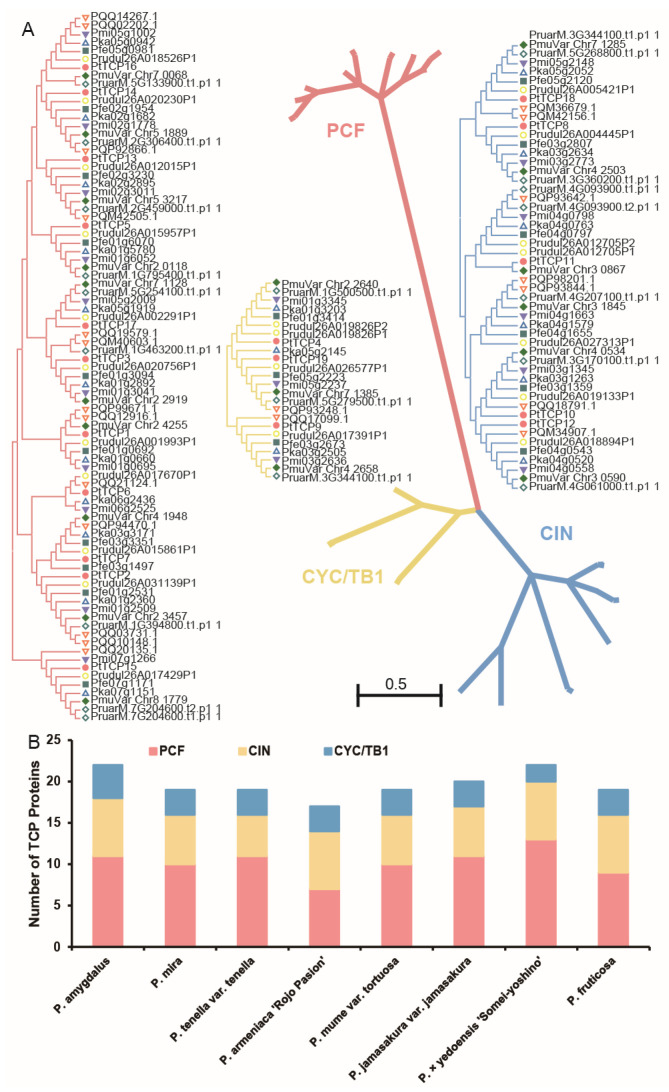
Phylogenetic analysis of the *TCP* genes in eight *Prunus* species. (**A**) Phylogenetic analysis of the TCP proteins; (**B**) the number of TCP proteins identified in the three groups.

A phylogenetic tree was constructed using TCP genes in eight *Prunus* species to examine the phylogenetic relationships of *TCP* genes in the *Prunus* species. Based on the phylogenetic tree, the TCP family was divided into PCF, CIN, and CYC/TB1 groups (Figure 2). Also, a phylogenetic tree was constructed by combining TCPs from *P. tenella* var. *tenella*, and *O. sativa* and *A. thaliana* also support the above classification (Figure S2). Among 157 TCPs, 82, 51, and 24 TCP transcription factors were divided into the PCF, CIN, and CYC/TB1 subgroups, respectively. Among all *Prunus* species, the PCF subfamily has the highest number of members (43.8–52.6%), while the CYC/TB1 subfamilies have the lowest numbers (12.5–20.0%).

### 3.3. Collinearity Analysis of TCP Genes

The collinearity analysis showed that most of the *TCPs* are segmentally duplicated genes (76.3%), followed by dispersed genes (23.7%) (Table S2). Only two tandem genes were found in *P. amygdalus.* Those results indicate that segmental duplication was the main driving force behind the amplification of *TCPs* in the *Prunus* species.

**Figure 3:**
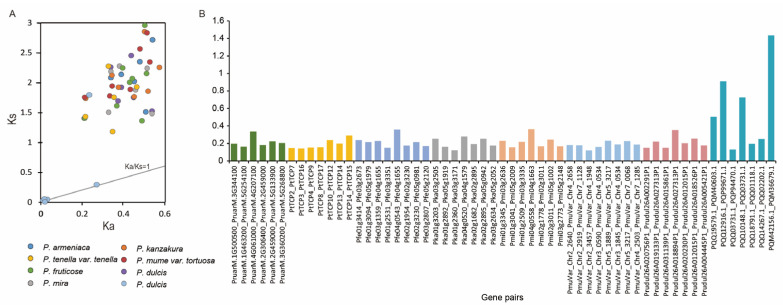
The Ka, Ks, and Ka/Ks values of *TCP* gene pairs in eight *Prunus* species. (**A**) The distribution of Ka and Ks values among *TCPs*; (**B**) the Ka/Ks values of *TCP* gene pairs.

The Ka (non-synonymous substitution)-to-Ks (synonymous substitution) ratio for each pair of paralogous genes was calculated for a closer look at the rate of *TCP* gene evolution in eight *Prunus* species. In this study, the Ks values of *P.* × *yedoensis* ‘Somei-yoshino’ gene pairs were mainly distributed between 0.009 and 1.798, whereas those of other *Prunus* species were between 1.0 and 3.0 (Figure 3A). The Ka values of most *P.* × *yedoensis* ‘Somei-yoshino’ gene pairs were less than 0.03, except PQQ12916.1_PQP99671.1 (0.272) and PQQ03731.1_PQP94471.1 (0.235). The Ka values of other plum plants were mostly between 0.2 and 0.6. In addition, the Ka/Ks value of other gene pairs was less than 1, except PQM42156.1_PQM36679.1 (1.436), indicating that PQM42156.1 and PQM36679.1 genes may be subjected to positive selection (Figure 3B).

**Figure 4:**
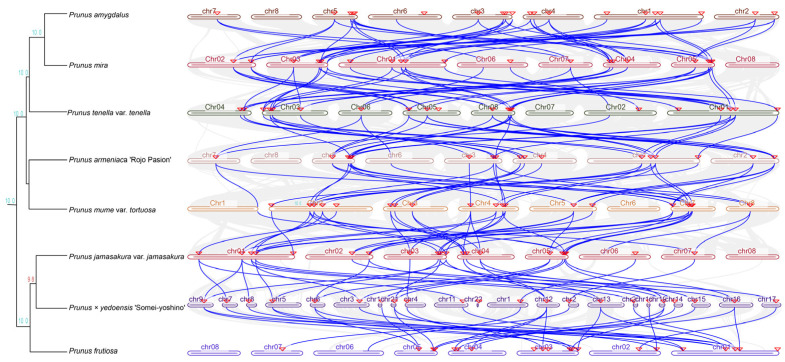
Phylogenetic tree and collinearity analysis of *TCP* genes in eight *Prunus* species. The triangle indicates the location of the gene, the chr represents the chromosome, and the blue line represents the *TCP* homologous gene.

Subsequently, a phylogenetic tree of eight *Prunus* species was constructed, and the genome collinearity of TCPs within the *Prunus* genus was determined based on the evolutionary relationships among the various species. The findings indicated significant collinearity across different *Prunus* species (Figure 4). The number of orthologous pairs in the *Cerasus* subgenus (*P. jamasakura* var. *jamasakura* vs. *P.* × *yedoensis* ‘Somei-yoshino’ and *P.* × *yedoensis* ‘Somei-yoshino’ vs. *P. fruticosa*) was 31 each. Further, 29 orthologous pairs were found between *P. mume* var. *tourtosa* and *P. armeniaca* ‘Rojo Pasion’ (*Prunus* subgenera). For the *Amygdalus* subgenus, 40 and 41 orthologous pairs were found in *P. amygdalus* vs. *P. mira* and *P. mira* vs. *P. tenella* var. *tenella*, respectively.

### 3.4. Conserved Motif and Domain Analysis of TCPs

**Figure 5:**
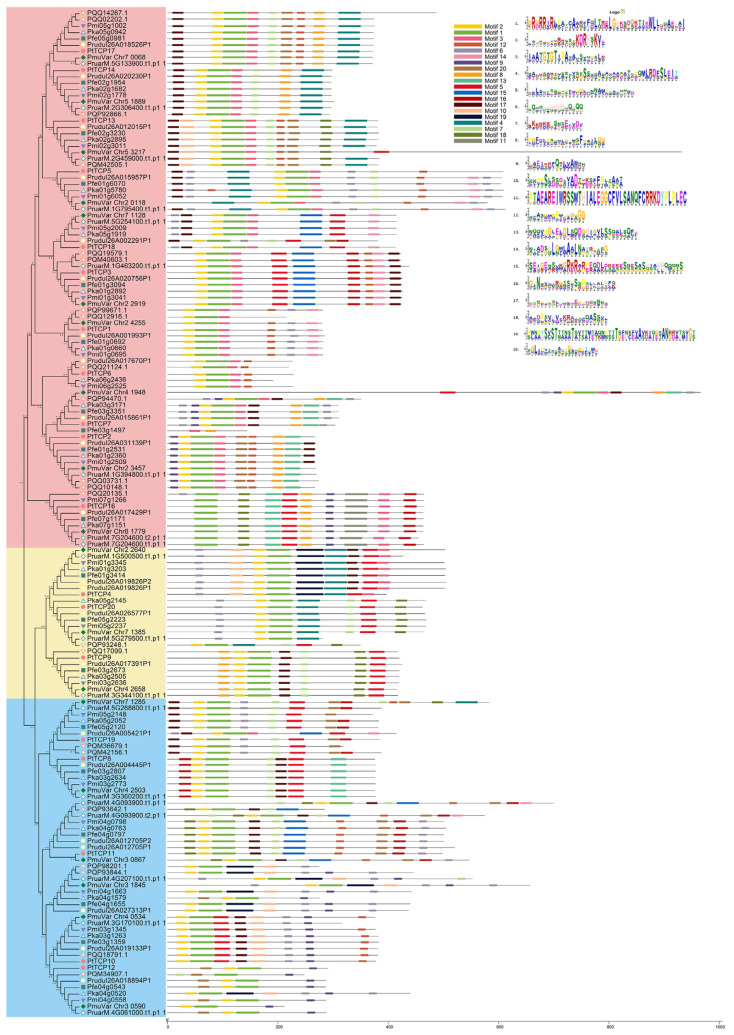
Analysis of conserved motifs in TCP proteins from eight *Prunus* species. Colored boxes represented different conserved motifs with different sequences and sizes. The overall height of each stack represents the degree of conservation at this position, whereas the height of the individual letters within each stack indicates the relative frequency of the corresponding amino acids.

All TCPs displayed the TCP domain, characterized by a basic helix–loop–helix structure (Figure S3). A total of 20 conserved motifs of 154 TCPs were analyzed to understand their structural characteristics (Figure 5). The motifs owned or shared by most members of the gene family may be an integral part of this gene family and have important functions or structures. Although the conserved motifs of TCPs differed in composition, all TCP proteins contained motif 1 and motif 23. In addition, different conserved motifs were present in different subfamilies. For example, motif 11 existed only in some members of the PCF subfamily, while motif 19 was exclusive to certain members of the CYC/TB1 subfamilies. Most members of the PCF subfamily contained motif 3.

### 3.5. Prediction of cis-Elements in the Promoter of TCPs

**Figure 6:**
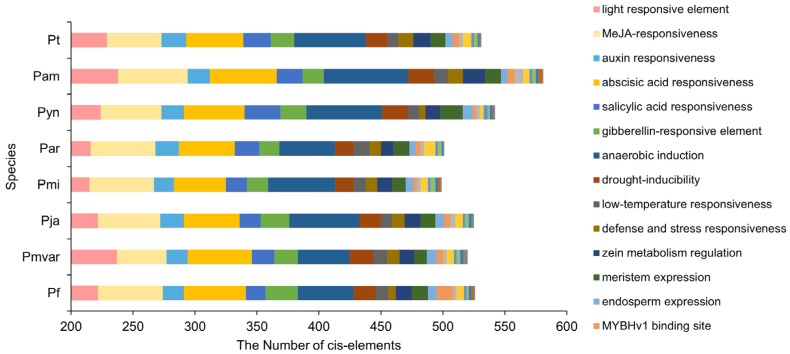
Distribution of cis-acting elements in the promoters of TCPs in eight *Prunus* species. Different colored squares show different cis-acting elements in the promoter. Pt: *Prunus tenella* var. *tenella*; Pam: *Prunus amygdalus*; Par: *Prunus armeniaca* ‘Rojo Pasion’; Pf: *Prunus fruticose*; Pja: *Prunus jamasakura* var. *jamasakura*; Pmi: *Prunus mira*; Pmvar: *Prunus mume* var. *tortuosa*; Pyn: *Prunus* × *yedoensis* ‘Somei-yoshino’.

PlantCARE was used to predict cis-acting elements in the promoter region of TCP genes (2000 bp upstream of the transcription start site) to explore the transcriptional regulation of TCPs (Figure 6). The findings indicate that 4225 cis-acting elements were identified. The number of cis-acting elements of the TCP promoters in eight *Prunus* species was variable (Figure 6). Among these, *P*. *amygdalus* contained the largest number of cis-acting elements (581), followed by *P.* × *yedoensis* ‘Somei-yoshino’ (542). These cis-acting elements primarily included the following: (1) light response-related elements, with an average of 11.5 elements per gene; (2) biological and abiotic stress response-related elements, such as drought inducibility (0.9 elements per gene), low-temperature responsiveness (0.5 elements per gene), defense and stress responsiveness (0.5 elements per gene), wound-responsive element (0.1 elements per gene), and anaerobic induction (2.7 elements per gene); (3) hormone response-related elements, such as abscisic acid (2.4 elements per gene), Methyl Jasmonate (MeJA) (2.5 elements per gene), auxin (0.9 elements per gene), gibberellin (1.0 elements per gene), salicylic acid (1.0 elements per gene); (4) development- and-tissue-specificity-related elements, including meristem expression (0.6 elements per gene), zein metabolism regulation (0.7 elements per gene), endosperm expression (0.3 elements per gene), circadian control (0.3 elements per gene), differentiation of the palisade mesophyll cells (0.1 elements per gene), seed specificity (0.1 elements per gene), and flavonoid biosynthesis (0.1 elements per gene) (Figure 6).

### 3.6. Expression of PtTCP Under Low Cold Stress

**Figure 7:**
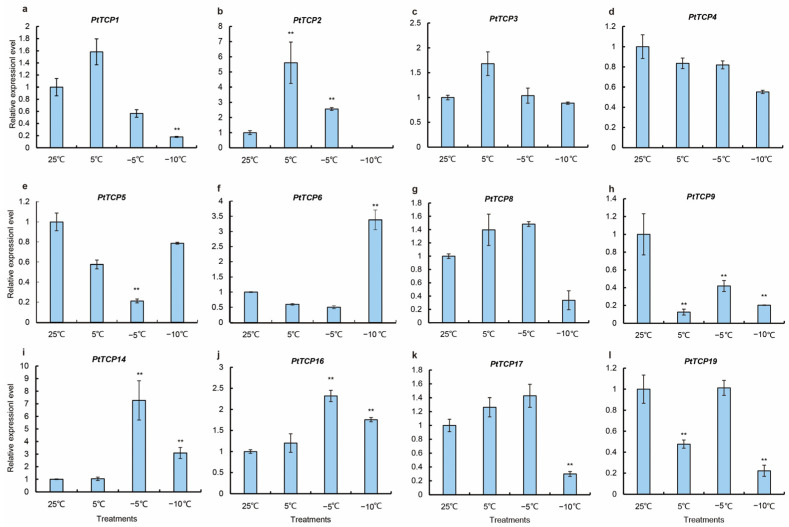
The expression patterns of 12 *PtTCPs* at different temperatures (25, 5, −5, and −25 °C) as revealed by qRT-PCR. The mean values were from three independent biological replicates. The data were statistically analyzed using Student’s *t*-test (** *p* < 0.01). (**a**–**l**) The relative expression levels of *PtTCP1*, *PtTCP2*, *PtTCP3*, *PtTCP4*, *PtTCP5*, *PtTCP6*, *PtTCP8*, *PtTCP9*, *PtTCP14*, *PtTCP16*, *PtTCP17*, and *PtTCP19* at different temperatures.

The expression profiles of *PtTCPs* were assessed after exposure to different temperatures (25 °C, 5 °C,−5 °C, and −10 °C) to investigate their expression under cold stress. The differential expression patterns of 12 *PtTCPs* under cold stress were detected (Figure 7). The expression levels of *PtTCP2*, *PtTCP14*, and *PtTCP16* initially increased and then decreased. The expression of *PtTCP1*, *PtTCP8*, and *PtTCP17* was inhibited, but the expression of *PtTCP6* increased at –10 ℃. Compared with the control, the expression levels of *PtTCP3* and *PtTCP4* remained unchanged.

### 3.7. Subcellular Localization of PtTCP9 Proteins

**Figure 8:**
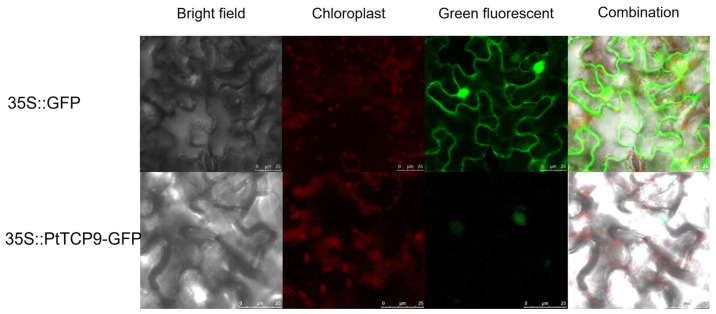
Subcellular localization of PtTCP9 proteins. Green fluorescent, green fluorescent signals; Bright field, bright field signals; Chloroplast, Chloroplast fluorescence signals; Combination, different fluorescent superimposed signals. Red fluorescence indicates chloroplasts, and grey images are bright field.

The constructed *pCambia1300-PtTCP9-GFP* vector was transiently transfected into *N*. *benthamiana* leaves to verify the subcellular position of PtTCP9. The results of confocal laser microscopy reveal that the green fluorescence signal displayed in the lower epidermal cells of *N. benthamiana* leaves transformed with the empty pCAMBIA1300-GFP vector was detected throughout the whole cell (Figure 8). In contrast, the green fluorescence signal in pCAMBIA1300-PtTCP9-GFP transgenic leaves was detected exclusively in the nucleus. These results indicate that PtTCP9 is a transcription factor localized in the nucleus, consistent with previous findings.

## 4. Discussion

TCP transcription factors play an essential role in the growth and development of plants as well as in responses to abiotic stresses. The genome-wide analysis of TCP has been performed in a large number of species: 27 in rice [28], 23 in *Andrographis paniculat* [29], and 23 in *Robinia pseudoacacia* [30], respectively. In Rosaceae, the number of TCP family members in different subfamilies varied: 52 in *Malus domestica*, 34 in *Pyrus bretschneideri*, 18 in *Rosa chinensis*, 19 in *Fragaria vesca*, 19 in *P. mume*, 17 in *Rubus occidentalis*, 20 in *P. persica*, and 14 in *P. avium* [31,32]. *M. domestica* and *P. bretschneideri* underwent whole-genome duplication (WGD) 3–4 billion years ago, which led to an increase in their chromosome numbers and may explain the higher number of TCP genes in these species [33,34]. The number of TCP genes in *Prunus* was similar (17–22), probably because *Prunus* plants did not experience WGD events.

Based on the phylogenetic analysis, the TCP proteins were classified into the PCF, CIN, and CYC/TB1 groups. Among these, the PCF subfamily contained the maximum number of genes (52.2%), and the CYC/TB1 group contained the least (15.3%). These proportions were different from the observations in *O. sativa* [27], *A. thaliana* [35], and Alfalfa [36], but were similar to those in *Melilotus albus* [37].

The results of motif analysis indicate that the types and quantities of motifs among the three classes significantly differed. Each class had its own unique motif, as well as a common motif among the three classes. Motif 1 was present in all *TCP*s. This further confirmed that the differences in motifs between different groups may be the main reason for their functional differences.

Previous studies have reported that most TCP proteins in other plants are located in the nucleus [28,29,30,35,36]. In this study, subcellular localization prediction results reveal that most TCPs were located in the nucleus. The experiment showed that the PtTCP9 protein was located in the nucleus. It is speculated that this family of genes mainly plays a regulatory role as TFs in the nucleus.

The differences among TCP proteins may be related to the involvement of different TCP genes in various processes such as organ development, signal transduction, and stress response [38,39,40,41,42]. Analysis of the subsequence of the *TCP*s promoter revealed that the promoter of *TCP*s contains several light-, low temperature-, and hormonal-responsive elements, such as abscisic acid and jasmonic acid, similar to the distribution type of the action elements of the promoter in the TCP gene family of *P. persica* [43].

The *TCP* genes have been reported to regulate abiotic stress in many plants. In *Sorghum bicolor*, the expression level of *SbTCP7* was upregulated under drought stress [44]. *HrTCP20* can improve the drought resistance of sea buckthorn by mediating JA signaling pathway in *Hippophae rhamnoides* [45]. In *Rosa Chinensis*, the expression of *RcTCP2* gradually increased under salt treatment, whereas the expression of *RcTCP6*, *8*, *12*, and *13* decreased [32]. Overexpression of the *Phyllostachys edulis PeTCP10* gene enhanced the salt tolerance of transgenic plants in the vegetative growth stage and increased the salt sensitivity in the germination and seedling stage [46]. Moreover, the *TCP* gene has been reported to be involved in regulating cold tolerance. *OsPCF6* and *OsTCP21* were regulated by miR319 and can reduce the frost cold resistance in *O. sativa* [42]. The gene expression analysis showed that the expression levels of *PtTCP6*, *PtTCP614*, and *PtTCP16* increased, whereas those of *PtTCP5*, *PtTCP8*, *PtTCP17*, and *PtTCP19* decreased under cold treatment. These results indicate that the functions of *PtTCPs* were varied, and several *PtTCPs* may participate in the response of *P. tenella* var. *tenella* to cold stress.

## 5. Conclusions

This study analyzed the TCP gene family in *Prunus* in terms of physical and chemical properties, subcellular location, phylogenetic relationship, gene structure, chromosomal location, cis-regulatory element, and response to cold stress under gene overexpression. The expression of four genes (*PtTCP2*, *PtTCP6*, *PtTCP14*, and *PtTCP16*) increased under cold stress. Future studies should focus on analyzing the biological functions of these genes in *P. tenella* var. *tenella* in response to cold stress and exploring their potential as excellent genetic resources for developing cold-resistant varieties of *P. tenella* var. *tenella*.

## Figures and Tables

**Table 1:** Number of *TCPs* in eight *Prunus* species.

Subgenus	Species Name	Chromosome Number	Genome Size (Mb)	Genome Protein Number	Number of TCP Genes	Number of TCP Proteins	Proportion of TCP Proteins (%)
Amygdalus	*P. tenella* var. *tenella*	8	220.3	32,088	19	19	0.059
	*P. amygdalus*	8	220.7	27,984	19	22	0.071
	*P. mira*	8	242.8	28,519	19	19	0.067
Cerasus	*P. jamasakura* var. *jamasakura*	8	375.3	26,986	20	20	0.074
	*P. fruticosa*	8	249.2	28,587	19	19	0.070
	*P.* × *yedoensis* ‘Somei-yoshino’	8	299.5	41,294	22	22	0.053
Prunus	*P. armeniaca* ‘Rojo Pasion’	8	251.3	40,067	17	19	0.045
	*P. mume* var. *tortuosa*	8	237.8	29,706	19	19	0.067

## Data Availability

The original contributions presented in the study are included in the article/Supplementary Material, further inquiries can be directed to the corresponding authors.

## Supplementary Materials

The following supporting information can be downloaded at: https://www.mdpi.com/article/10.3390/genes15111443/s1, Table S1. The primer sequences of genes used in qRT-PCR analysis; Table S2. Characteristics of the members of the TCP gene family of eight *Prunus* species; Table S3. Ka/Ks analysis of the TCP homologous gene pairs from eight *Prunus* species. ‘WGD’ or ‘segmental’ means that the gene might arise from Whole Genome Duplication or Segmental Duplication. ‘Dispersed’ means that the gene might arise from transposition; Figure S1. The characteristics of *Prunus tenella* var. *tenella.* (A) Leaf and flower; (B) fruit; (C) nutlet; Figure S2. Phylogenetic analysis of the TCP genes from *Arabidopsis thaliana*, *Prunus tenella* var. *tenella*, and *Oryza sativa*; Figure S3. Multiple sequence alignment of the TCP domain. Multiple sequence alignment was conducted with DNAMAN.
