# Supplementary figures and images for: Genome-Wide Identification and Characterization of TCP Genes in Eight Prunus Species and Their Expression Patterns Under Cold Stress in P. tenella var. tenella
Source: Genes (Basel). 2024 Nov 8;15(11):1443. doi: 10.3390/genes15111443 (PMC11593384; doi:10.3390/genes15111443)

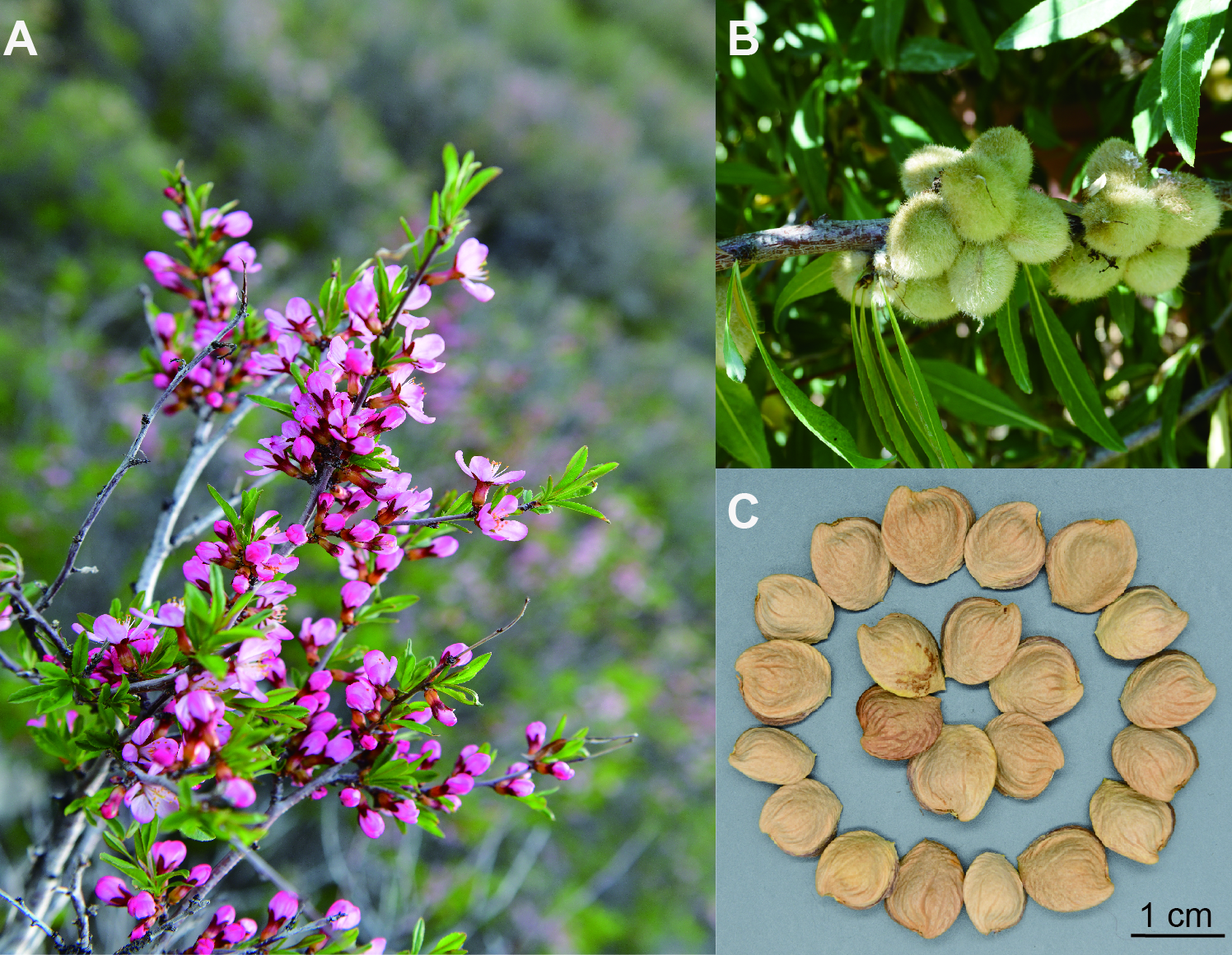

Supplement: Supplementary file 1 [file genes-15-01443-s001.zip › 10.29/Figure S1.jpg]

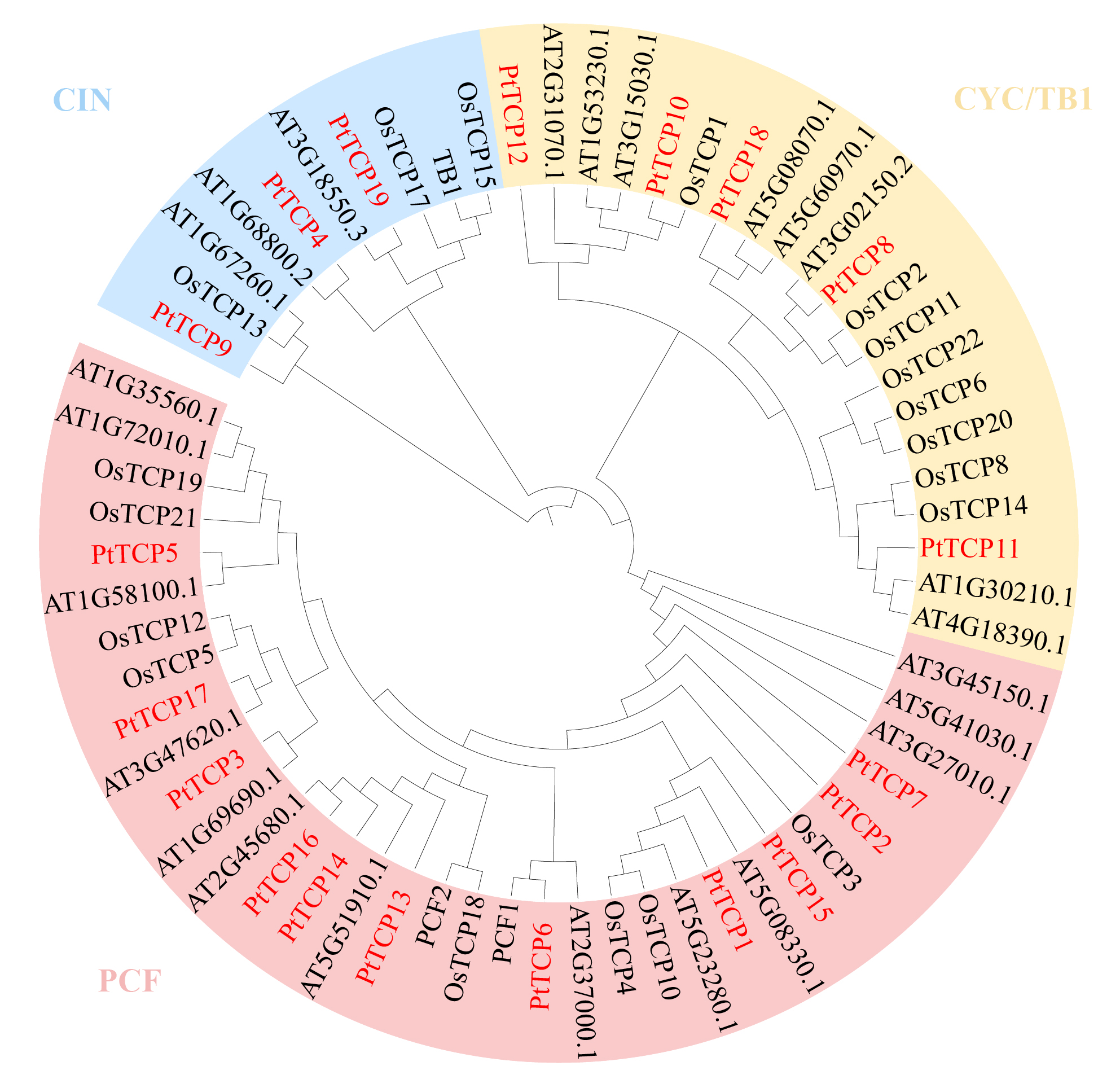

Supplement: Supplementary file 1 [file genes-15-01443-s001.zip › 10.29/Figure S2.jpg]

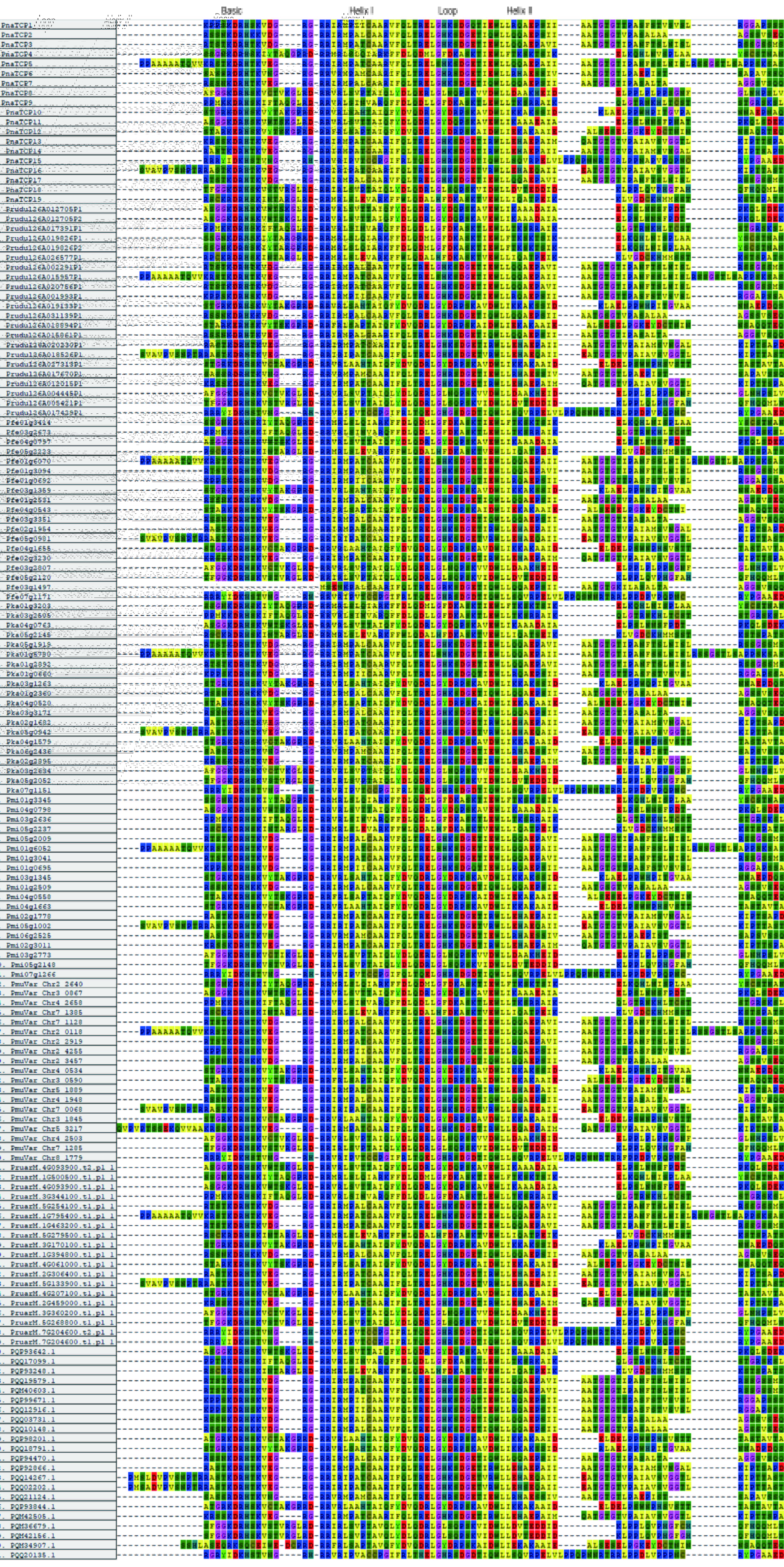

Supplement: Supplementary file 1 [file genes-15-01443-s001.zip › 10.29/Figure S3.jpg]
